# A Brief Intervention for Improving Alcohol Literacy and Addressing Harmful Alcohol Use Among Women Attending an Australian Breast Screening Service (Health4her): Protocol for a Hybrid Effectiveness-Implementation Trial

**DOI:** 10.2196/44867

**Published:** 2023-03-30

**Authors:** Jasmin Grigg, Victoria Manning, Darren Lockie, Michelle Giles, Robin Bell, Peta Stragalinos, Chloe Bernard, Isabelle Volpe, Christopher J Greenwood, Liam Smith, Peter Bragge, Dan I Lubman

**Affiliations:** 1 Turning Point Eastern Health Melbourne Australia; 2 Monash Addiction Research Centre Eastern Health Clinical School Monash University Melbourne Australia; 3 Maroondah BreastScreen Eastern Health Melbourne Australia; 4 School of Public Health and Preventive Medicine Monash University Melbourne Australia; 5 School of Psychology Deakin University Geelong Australia; 6 Centre for Adolescent Health Murdoch Children’s Research Institute Melbourne Australia; 7 BehaviourWorks Australia Monash University Melbourne Australia

**Keywords:** alcohol, alcohol literacy, alcohol brief intervention, breast cancer, women’s health, randomized controlled trial, hybrid effectiveness-implementation trial, protocol

## Abstract

**Background:**

Alcohol consumption is a major modifiable risk factor for female breast cancer, even in small amounts. However, awareness of this risk remains low. National breast screening programs are uniquely positioned to provide timely and targeted health information and behavior change strategies to improve alcohol literacy and reduce consumption. A breast screening service is a novel health care setting for brief alcohol intervention, with the potential for extensive reach.

**Objective:**

This study aimed to conduct a formative evaluation with breast screening service consumers to understand the need for, and acceptability of, brief alcohol intervention in the breast screening setting and collaboratively design a brief alcohol intervention (Health4Her); to test the effectiveness of Health4Her in improving knowledge of alcohol as a breast cancer risk factor (primary outcome), improving alcohol literacy, and reducing consumption among women attending a breast screening service; and to examine the implementation strategy through process evaluation.

**Methods:**

This was a hybrid type II effectiveness-implementation trial comprising a randomized controlled trial (RCT) alongside a mixed methods program evaluation guided by applicable elements of the Reach, Effectiveness, Adoption, Implementation, and Maintenance framework and Consolidated Framework for Implementation Research. Formative evaluation comprised a retrospective analysis of alcohol consumption data (n=49,240), a web-based survey (n=391), and focus groups and interviews (n=31) with breast screening service consumers. Women attending routine mammography, drinking at any level, were recruited to the single-site, double-blind RCT (n=558), and completed a baseline assessment before randomization (1:1) to receive Health4Her (alcohol brief intervention + lifestyle information) or control (lifestyle information) via animation on an iPad. Follow-up assessments were performed 4 and 12 weeks after randomization. The process evaluation included evaluation of trial administrative data, participant quantitative (n=497) and qualitative feedback (n=30), and site staff qualitative feedback (n=11).

**Results:**

This research was funded in March and May 2019. Data collection for the formative evaluation and trial recruitment occurred between January and April 2020 and February and August 2021, respectively, with finalization of follow-up data collection in December 2021. Quantitative process evaluation data were collected during trial implementation, and collection of participant and staff feedback was finalized in December 2021. Results of the retrospective analysis of alcohol consumption data from breast screening service consumers is anticipated to be published in March 2023 and the results of the RCT to be published in March 2023.

**Conclusions:**

This study is anticipated to generate new substantial knowledge on the alcohol consumption and literacy needs of women attending breast screening and the extent to which these can be addressed using a novel, tailored brief alcohol intervention. The study design permits the evaluation of the effectiveness and implementation of Health4Her to predict and facilitate uptake in breast screening services.

**Trial Registration:**

ClinicalTrials.gov NCT04715516; https://clinicaltrials.gov/ct2/show/NCT04715516

**International Registered Report Identifier (IRRID):**

RR1-10.2196/44867

## Introduction

### Background

Globally, alcohol use is a major modifiable risk factor for breast cancer in women, accounting for 4.4% of breast cancer cases and 10% of breast cancer deaths [1,2]. Meta-analyses demonstrate a dose-response relationship between alcohol and breast cancer [3-5] with the relative risk of breast cancer increasing by 4%, 23%, and 61% for light, moderate, and heavy alcohol consumption, respectively [3]. Lifetime and current alcohol consumption have been found to contribute to an increased risk of breast cancer [6-8]. In particular, alcohol consumption after the age of 40 years is strongly and independently associated with breast cancer risk [7]. Among women with a history of breast cancer, alcohol intake of <1 standard drink per day has been associated with an increased risk of breast cancer recurrence or development of a second primary breast cancer [9].

Despite strong evidence, awareness of the alcohol–breast cancer link has remained surprisingly low [10]; recent Australian and international estimates indicate that only 16% to 20% of the population accurately identifies alcohol consumption as a risk factor for breast cancer [11,12]. In Australia, per capita alcohol consumption is declining [13]; however, risky alcohol consumption (based on 2009 Australian Alcohol Guidelines, consuming >2 Australian standard drinks per day) has remained stable among women aged ≥40 years and has even increased among women aged between 50 and 69 years [14]. Furthermore, a prospective cohort study of Australian women with newly diagnosed first episode of invasive breast cancer found that the proportion of women drinking at the time of diagnosis and 2 years after diagnosis remained stable at 71%, with 1 in 6 women continuing to drink in excess of the national alcohol guidelines that were in place at the time (ie, >2 standard drinks per drinking day) after breast cancer diagnosis [14,15]. Despite this, alcohol consumption among middle- to older-age individuals is often underrecognized as an area of concern [16,17] and has largely been absent from breast cancer prevention strategies and health promotion.

Internationally, computerized brief alcohol interventions (ie, feedback on personal drinking levels compared with age and gender norms, alongside information about alcohol risks and harms) have received strong empirical support as a simple, cost-effective method of addressing alcohol consumption and reducing related harm in primary care populations [18]. Computerized brief alcohol interventions offer a low-cost, labor- and time-efficient approach that has the potential to overcome some of the issues with implementation in busy health care environments [19,20]. Meta-analyses show that a single computerized intervention session is effective in reducing alcohol consumption and improving health-related knowledge, attitudes, and intentions [18,21] and has the potential for wide reach.

Population-based screening programs can provide a window of opportunity to receive relevant targeted health information. Approximately 43 million women participate in population breast screening programs annually in Australia, the United States, and the United Kingdom. An intervention offered within an already established national breast screening program, which could provide information on alcohol and breast cancer risk alongside behavioral change strategies, has the potential to improve women’s alcohol literacy, reduce alcohol consumption, and have extensive reach. Some recent research suggests that integrating brief alcohol interventions into breast screening services is perceived by clients as acceptable [20,22]; however, the effectiveness of such an approach is yet to be established.

### Objectives

The aims of this research were to (1) conduct a formative evaluation with breast screening service consumers to understand the need for, and acceptability of brief alcohol intervention in the breast screening setting and collaboratively design a brief alcohol intervention (Health4Her) with women in this setting; (2) test the effectiveness of Health4Her in improving knowledge of alcohol as a breast cancer risk factor (primary outcome), improving alcohol literacy, and reducing consumption among women attending a breast screening service; and (3) examine the implementation strategy through process evaluation to accelerate the translation of this research into practice.

## Methods

### Study Design

**Figure 1:**
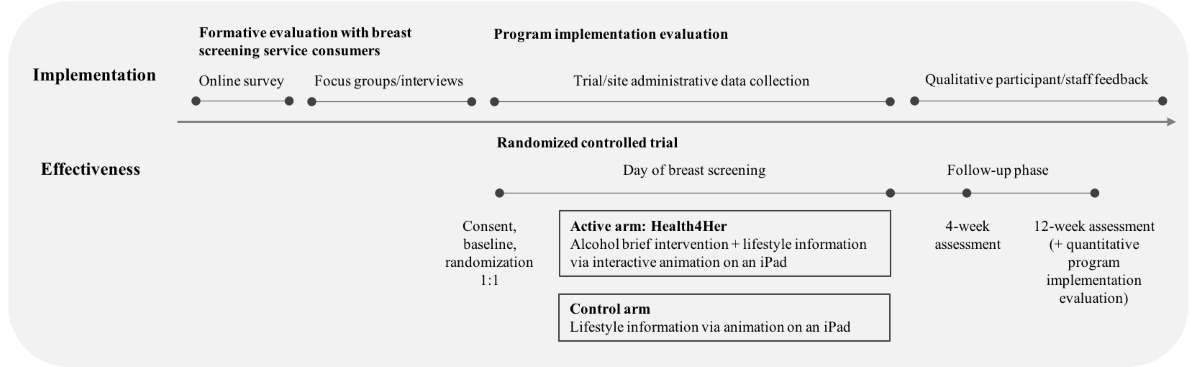
Study design.

This was a hybrid type II effectiveness-implementation trial [23,24] comprising a randomized controlled trial (RCT) alongside a mixed methods program evaluation (Figure 1), guided by applicable elements of the Reach, Effectiveness, Adoption, Implementation, and Maintenance (RE-AIM) framework and Consolidated Framework for Implementation Research (CFIR) [25,26]. A mixed methods quantitative and qualitative formative evaluation comprised a retrospective analysis of cross-sectional alcohol consumption data from a large sample of breast screening service consumers, a web-based survey, and focus groups and interviews with subsets of breast screening service consumers. The single-site, parallel group, double-blind RCT was designed to evaluate whether the Health4Her intervention is effective in improving knowledge of alcohol as a breast cancer risk factor, improving alcohol literacy, and reducing consumption among women attending a breast screening service. A mixed methods quantitative and qualitative process evaluation examined the implementation strategy via the evaluation of trial and site administrative data, quantitative and qualitative feedback from trial participants, and qualitative feedback from breast screening site staff. The RCT protocol followed the Standard Protocol Items: Recommendations for Interventional Trials guidelines (Multimedia Appendix 1) [27] and the Template for Intervention Description and Replication checklist (Multimedia Appendix 2) [28].

### Formative Evaluation

#### Setting

Researchers conducting the formative evaluation were based at Turning Point, a national addiction treatment and research center in Melbourne, Australia. To collect qualitative data from breast screening service consumers, focus groups were initially conducted on-site and replaced with telephonic interviews conducted from the end of March 2020 to comply with social distancing requirements owing to the COVID-19 pandemic.

#### Participants

The first component of the formative evaluation comprised a retrospective analysis of deidentified cross-sectional alcohol and sociodemographic data (collected from May 2010 to November 2019) from 49,240 women who are members of Lifepool [29], a large convenience sample of women living in Victoria, Australia, who are recruited predominantly through the state-wide BreastScreen program. For the web-based survey, 391 participants were recruited through an email invitation circulated to randomly sampled batches of Lifepool members, stratified by age, based on an anticipated response rate of 25% (total invitations e-mailed, n=1412). Women who participated in the web-based survey and were living in the Melbourne region were invited to register for participation in a focus group or interview at the end of the survey. In addition, purposive sampling was employed to recruit women living in the Melbourne region who reported drinking 3 to 4 (or more) standard drinks, 3 to 4 (or more) days per week (based on the data collected at cohort entry), to participate in a focus group or interview (additional invitations, n=199; focus group or interview participation, n=31). As with all components of this study, women with or without a history of breast cancer were able to participate.

#### Data Collection

For the retrospective analysis of Lifepool data, there were data available on participants’ typical and heavy alcohol consumption patterns over the previous 12 months (eg, *“*In the last 12 months, how often did you have an alcoholic drink of any kind?” and “In the past 12 months, how often have you had more than 4 standard drinks in a day?”) [30]. Sociodemographic (ie, age, education, country of birth, and identifying as being of Aboriginal and/or Torres Strait Islander descent) and health-related (ie, hours per week of moderate-intensity physical activity, BMI, menopausal status, breast cancer diagnosis, first-degree relative breast cancer history, and smoking status) variables were also examined. Postcode data were used to derive the geographical remoteness [31] and relative socioeconomic advantage [32] variables, as defined by the Australian Bureau of Statistics.

The web-based survey, developed by the research team and tested with a convenience sample of women employees at Turning Point, collected demographic information (eg, age and education) and data on women’s knowledge of breast cancer risk factors, breast cancer information sources, the acceptability of receiving cancer risk information in various health settings, and alcohol consumption patterns. Participants’ typical and heavy alcohol consumption patterns over the previous 12 months was measured using quantity-frequency items (eg, *“*In the last 12 months, how often did you have an alcoholic drink of any kind?” and “On a day that you have an alcoholic drink, how many standard drinks do you usually have?”) [30]. A visual standard drinks guide was provided alongside alcohol consumption questions to increase response accuracy and equivalence across participants.

Qualitative focus groups were facilitated by a senior research assistant, IV, and cofacilitated by at least one other researcher (ie, JG, CB, or PS). IV conducted the interviews. The semistructured focus group and interview schedule gathered information regarding women’s views in relation to breast cancer risk, alcohol consumption, other lifestyle factors, information needs, and attitudes toward receiving health information in different health settings. Women participated in a collaborative design activity where they provided feedback on and generated ideas for the design of the brief alcohol intervention prototype (eg, format, alcohol messaging), as well as the planned research procedures.

#### Data Analyses

Summary data from the retrospective analysis of Lifepool data and the web-based survey were expressed as counts and proportions or means and SDs. For the retrospective analysis of Lifepool data, sociodemographic and health-related characteristics were compared among women drinking within or exceeding the limits set by national alcohol guidelines for weekly and daily consumption. Associations were explored using logistic regression (categorical variables, dummy coded) and linear regression (continuous variables); in separate models, each characteristic dependent variable was regressed onto the alcohol guideline independent variables. Analyses were performed using Stata (version 17; StataCorp LLC). A *P* value <.001 (2-sided) was used as the level of significance for statistical analyses.

For qualitative data, focus groups and interviews were audio-recorded and transcribed verbatim by a professional transcribing company and organized using NVivo 20 (QSR International) according to the relevant domains of the RE-AIM and CFIR frameworks. Deductive thematic analysis was conducted using the framework method [33].

#### Reimbursement

To optimize participation in the formative evaluation, there was an incentive of an Aus $1 (US $0.69) donation to breast cancer research for every web-based survey completed and an Aus $10 (US $6.91) donation for focus group or interview participation.

### RCT Protocol

#### Setting

This RCT was conducted at Maroondah BreastScreen, an Eastern Health Service and part of the Victorian National Breast Cancer Screening Program in Melbourne, Australia.

#### Participants and Eligibility Criteria

Women who underwent routine mammography on Tuesdays and Fridays between February and August 2021 were invited to participate in the study after their appointment. Women received their usual appointment reminder emails with additional information to inform them about the study in advance. Study posters were displayed in the waiting and screening rooms, and verbal information about the study was provided by the radiographers and reception staff. A text message reminder informing women about the study the day before their appointment was introduced, in addition to the email, in May 2021. The inclusion and exclusion criteria are presented in Textbox 1. Women drinking at any level of alcohol consumption (including women who do not drink) were eligible to participate. This approach served to negate any stigma and potential unblinding that may occur by including only women who consume alcohol or drink at risky levels.

**Eligibility criteria.**

**Inclusion criteria**

- Female
- Attending routine mammography
- Aged ≥40 years
- English as a first language or fluent
- Regular access to a telephone
- Able to provide informed consent to participate
- Any level of alcohol consumption (including women who do not drink)

**Exclusion criteria**

- Hearing impairment sufficient to prohibit a telephone interview
- Pregnancy (also an exclusion from screening)
- Unable to read or comprehend English at a level to provide informed consent or receive the brief intervention

#### Randomization and Blinding

Randomization was performed immediately after screening assessment, with the next consecutive allocation revealed on the protected spreadsheet. Participants were randomly assigned to the intervention or control group at a 1:1 allocation ratio. Randomization used standard computer-generated permuted blocks of a variable-size scheme. A randomization list was generated at the start of the study by a data scientist and linked to a unique identification code. The data scientist generating the random allocation sequence played no other role in the study. The study was described as a trial of two types of women’s health promotion that minimized the trial’s alcohol focus and concealed allocation to the intervention arm. Researcher 1, who administered the corresponding intervention per a priori generated randomization list, was not blinded to trial allocation. Researcher 2, who performed the 4- and 12-week telephone assessments, was blinded to trial allocation throughout trial data collection and may only have been unblinded once quantitative program implementation evaluation feedback was sought from trial participants before closing the 12-week call (ie, data collection that did not require blinding). Given the nature and content of the intervention and the outcome assessments, emergency unblinding procedures were deemed unnecessary.

#### Interventions

##### Overview

Interventions were delivered on an iPad using a prototype of a brief eHealth intervention (ie, researcher-administered screening alcohol questions, intervention administered by the researcher for the participant to view alone). The brief eHealth intervention model was chosen to minimize disruption to the service workflow and the need for human resources to be implemented within the busy breast screening environment. Take-home study-branded earphones were provided to each participant to view the animation privately. The Qualtrics web-based survey platform that hosted the baseline alcohol questions and intervention tracked the time participants spent viewing the intervention, permitting oversight of the intervention completion.

##### Active Condition: Health4Her Alcohol Brief Intervention + Lifestyle Information

The active arm received 4 minutes of brief alcohol intervention and 3 minutes of lifestyle health promotion related to modifiable breast cancer risk factors (physical activity and maintaining a healthy weight). A take-home pamphlet summarizing the alcohol information presented during the intervention was provided along with a pamphlet on nutrition to maintain a healthy weight.

The Health4Her intervention was developed in accordance with brief alcohol intervention principles [34,35], applied behavior change approaches [36-39], and findings from our formative research with breast screening service consumers. The brief alcohol intervention comprised personalized feedback and comparison to gender and age drinking norms, negative-framed messaging around alcohol risks and harms, positive-framed messaging on the health benefits of reducing alcohol intake, and alcohol harm-reduction strategies (Table 1). There were 2 versions of the Health4Her intervention, whereby messages relating to personalized normative feedback varied depending on whether participants reported that alcohol use exceeded national alcohol guidelines.

**Table 1:** Examples of messages included in brief intervention.

Brief intervention and applied behavior change approach			Example of message received
**Personalized normative feedback**			
	Not exceeding Australian Alcohol Guidelines for weekly consumption	“You indicated that you are drinking within the recommended amount of 0 to 10 standard drinks per week. You’re doing a good thing for your health—the less you choose to drink, the lower your risk of alcohol-related harm.”	
	Exceeding Australian Alcohol Guidelines for weekly consumption	“You indicated that you are drinking above the recommended limit of 10 standard drinks per week. You are drinking more alcohol than three-quarters of Australian women your age, and the amount you are drinking is putting your health at risk.”	
Negative-framed messaging			“Did you know that alcohol is a carcinogen, meaning that it’s consumption can cause cancer in humans? And there’s now strong evidence that drinking alcohol increases your risk of breast cancer.”
Positive-framed (or gain-framed) messaging			“The less you choose to drink, the greater the immediate and longer-term health benefits.”
Alcohol harm-reduction strategies			“Here are some strategies to get you started: keep track of how much you’re drinking by checking the label of any bottle or can for the number of standard drinks it contains. Plan your alcohol-free days for the week ahead, and challenge yourself to add an extra alcohol-free day.”

##### Control condition: Lifestyle Information

The control arm received 3 minutes of lifestyle health promotion to increase knowledge of how to improve women’s health and reduce breast cancer risk, not inclusive of alcohol information. Lifestyle health promotion focused on physical activity and maintaining a healthy weight and was developed to be relevant to women attending breast screening services as 2 recognized modifiable breast cancer risk factors [40,41]. A take-home pamphlet on nutrition to maintain healthy weight was also provided.

#### Choice of Comparator

Participants in the control arm received lifestyle health information to reduce breast cancer risk through animation and a take-home pamphlet on nutrition to maintain a healthy weight. As confirmed through the formative evaluation, embedding brief alcohol intervention within lifestyle information offered to all women attending breast screening provided the opportunity to target harmful alcohol consumption in a discrete, nonstigmatizing way and increased Health4Her’s relevance for all women. As such, lifestyle information in the control condition was also provided in the intervention condition. This also served to maintain equivalence across the treatment conditions. Data from several studies suggest that alcohol assessment reactivity (ie, mere exposure to screening and baseline alcohol questions can prompt awareness and behavioral self-regulation) can bias trial results toward the null and lead to underestimation of the true effects of interventions [42,43]. While participants in the control arm were anticipated to experience some benefits from trial participation and alcohol assessments, it was expected that the effects on primary and secondary end points would be less than those observed for the intervention arm.

#### Outcomes

Primary and secondary outcomes of the RCT are detailed in Textbox 2.

**Trial outcomes.**

**Primary outcome**

- Change in the proportion of participants accurately identifying alcohol as a clear risk factor for breast cancer at 4-week postrandomization

**Secondary outcomes**

- Change in the proportion of participants drinking ≤10 standard drinks per week (ie, within current Australian Alcohol Guidelines) [44] at 4 week and 3 months postrandomization (14-day timeline follow-back [TLFB]) [45]
- Among participants who (1) have consumed any alcohol in the past 2 week and (2) drink >10 standard drinks per week at baseline: change in the proportion of participants drinking ≤10 standard drinks per week at 4 week and 3 months postrandomization (TLFB) [45]
- Change in alcohol consumption at 4 week and 3 months postrandomization (TLFB, Australian Institute of Health and Welfare alcohol frequency quantity items) [45-47]
- Among participants who (1) have consumed any alcohol in the past 2 week and (2) drink >10 standard drinks per week at baseline: change in alcohol consumption at 4 week and 3 months postrandomization (TLFB, Australian Institute of Health and Welfare alcohol frequency quantity items) [45-47]
- Health literacy (attitudes): change in participants’ attitudes regarding alcohol and breast cancer risk at 4-week postrandomization (adapted from previous literature) [48]
- Health literacy (knowledge): change in the proportion of participants accurately identifying (1) the amount of alcohol in an Australian standard drink; (2) the number of standard drinks in an average restaurant serve of red wine; (3) the maximum number of standard drinks per week recommended by current Australian Alcohol Guidelines (multiple-choice and open-ended questions, adapted from previous literature) [10] at 4-week postrandomization.
- Health literacy (access to health information): proportion of participants who have accessed health information on (1) alcohol harms, (2) alcohol and breast cancer risk, and (3) alcohol harm-reduction at 4-week postrandomization.
- Change in general health at 4 week and 3-month postrandomization (12-item Short-Form Health Survey) [49]
- Change in quality of life at 4 week and 3-month postrandomization (European Health Interview Survey-Quality of Life-single item) [50]

#### Informed Consent, Enrollment, and Participation

After the breast screening appointment, women who were interested in participating in or learning more about the study were introduced to the on-site researcher (researchers held, at a minimum, an honors degree in Psychology, Population Health, or related discipline), who provided a copy of the participant information and consent form, provided a verbal explanation of the trial (aims, procedures, risks, and benefits), and answered any participant questions (Table 2). Women were advised that they could withdraw from the study at any time and that their decision to participate or withdraw would not affect their relationship with Maroondah BreastScreen or Eastern Health. For women interested in participating in the study, the researcher confirmed their eligibility and obtained verbal or written informed consent (time-dependent), which included consent for the possible future use of participants’ deidentified data for related projects.

The researcher then conducted a brief baseline assessment and random allocation by manually administering the iPad to play the corresponding animation according to (1) randomization and (2) if allocated to the active arm, delivery of version the intervention consistent with the participant’s level of alcohol consumption. The study explanation, informed consent, screening, baseline assessment, and participation were brief, taking approximately 15 minutes per participant (Table 2).

**Table 2:** Standard Protocol Items: Recommendations for Interventional Trials schedule of enrollment, interventions, and assessments.

			Time point				
			Baseline (Maroondah BreastScreen)		Follow-up (telephone)		Follow-up (telephone)
			Day 1		4 weeks		12 weeks
**Enrollment**							
	Verbal information and provision of trial information sheet	✓					
	Informed consent (written or verbal; time-dependent)	✓					
	Eligibility and demographic information	✓					
	Randomization	✓					
**Intervention**							
	Active condition: Health4Her alcohol brief intervention + lifestyle information	✓					
	Control condition: lifestyle information	✓					
**Assessment^a^**							
	Knowledge assessment of breast cancer risk factors, including alcohol	✓		✓ ^b^			
	14-day timeline follow-back [45]	✓		✓		✓	
	Australian Institute of Health and Welfare alcohol frequency and quantity items [46,47]	✓		✓		✓	
	Assessment of alcohol literacy	✓		✓			
	European Health Interview Survey-Quality of Life (single item) [50]	✓		✓		✓	
	Short Form Health Survey [49]	✓		✓		✓	
	Assessment of physical activity [51] and fruit and vegetable consumption [52]	✓		✓		✓	

^a^Alcohol assessments were nested among general health and lifestyle questions to conceal alcohol focus.

^b^Primary outcome.

#### Follow-up and Lost to Follow-up

Follow-up assessments were conducted by researcher 2 via telephone calls (<15-minutes duration) at 4 and 12 weeks after randomization, wherein baseline measures were repeated (Table 2). Participants who could not be contacted after 5 attempts were deemed missing at that data collection time point. For participants lost to follow-up at 4 weeks, the researcher attempted contact at 12 weeks following the same procedure.

#### Schedule of Enrollment, Interventions, and Assessments

The schedule of enrollment, interventions and assessments is outlined in Table 2.

#### Reimbursement

Trial participants went into a draw to win one of the 10 Aus $100 (US $69.48) supermarket vouchers to optimize the follow-up response rate [42]. The participants received no direct financial or other consideration (ie, in kind contribution) to participate in this trial.

#### Outcome Measures

Outcome measures of the randomized controlled trial are shown in Table 3.

**Table 3:** Standard Protocol Items: Recommendations for Interventional Trials schedule of enrollment, interventions, and assessments.

Data collected				Method
**Screening**				
	Eligibility and demographic information		Brief standard demographic characteristics (eg, age and education) and inclusion and exclusion information collected through structured questions.	
**Primary outcome**				
	Knowledge assessment of breast cancer risk factors		Participants were asked to demonstrate their knowledge of breast cancer risk associated with 6 factors (eg, family history of breast cancer, physical inactivity, antiperspirant deodorant use, alcohol, processed meats, and excess weight) by selecting from a set of scaled-response options: (1) a clear breast cancer risk factor with strong, consistent evidence; (2) a possible risk factor with some evidence; (3) not a proven risk factor with too limited evidence to determine risk; or (4) do not know. Primary outcome was the proportion of participants identifying alcohol as a clear breast cancer risk factor at 4 weeks. Adapted from Cancer Australia’s 2018 definitions of breast cancer risk factors and previous research with breast screening service consumers [48,53].	
**Secondary outcomes**				
	**Alcohol^a^**			
		14-day TLFB^b^	Alcohol consumption over past 14 days assessed with TLFB [45]. TLFB also informed personalized feedback provided in the alcohol brief intervention.	
		AIHW^c^ alcohol frequency and quantity items	Past-month frequency of alcohol consumption and volume of alcohol consumption on a typical drinking day assessed with 2 items from the AIHW National Drug Strategy Household Survey [46,47].	
		Assessment of alcohol health literacy	Adapted from previous research with breast screening service consumers, participants were asked about (1) attitudes to alcohol and breast cancer risk [48]; (2) alcohol health knowledge (eg, amount of alcohol in a standard drink, number of standard drinks in an average restaurant serve of alcohol, maximum number of standard drinks recommended by current health guidelines) [10]; and (3) access to alcohol and breast cancer health information [54]. Questions were presented in multiple-choice, open-ended, or statement (ie, 5-point Likert scale from strongly agree to strongly disagree) formats.	
	**Health and well-being**			
		European Health Interview Survey-QOL^d^ single item	QOL rated as single item on 5-point Likert scale from very poor to very good [50].	
		SF-12^e^	SF-12 used as a brief measure of general health [49].	
		Physical activity duration [51,52] and consumption of vegetables and fruit [52]	Brief physical activity and vegetable and fruit intake questions included to ensure blinding of participants. Questions adapted from the Australian Bureau of Statistics and previous research [51,52].	

^a^Alcohol assessments were nested among general health and lifestyle questions to conceal the trial’s alcohol focus.

^b^TLFB: timeline follow-back.

^c^AIHW: Australian Institute of Health and Welfare.

^d^QOL: quality of life.

^e^SF-12: 12-item Short-Form Health Survey.

#### Data Collection and Management

Baseline data were collected by researcher 1 (on-site at Maroondah BreastScreen) and follow-up data were collected by researcher 2 (via telephone). A description of the trial schedule of the assessments can be found in Tables 2 and 3. An electronic case report form was completed for each participant using REDCap (Research Electronic Data Capture; Vanderbilt University) [55], hosted on a secure server and managed by Eastern Health IT Services with individual access via a secure login, and accessible only to approved members of the research team. REDCap stored screening and trial data, as well as identifiable data, including participant names and contact information. Identifiers were flagged in REDCap to provide additional protection for these data during data exports (ie, identifiers were automatically removed from all exported material generated by REDCap). Spreadsheets containing reidentifiable information (ie, for the purpose of conducting follow-up assessments; timeline follow-back data, which could not be collected using REDCap) were protected with unique passwords. Hardcopy data were stored and secured in a locked cabinet on-site at Turning Point. Upon completion of trial data collection, data were transferred from the electronic case report form and other source documents (eg, timeline follow-back spreadsheets) to Stata and R statistical software (R Foundation for Statistical Computing) packages for analyses. Protected health information (ie, participant name and contact information) was not exported. All data collected during this study will be retained by the research team for at least 5 years, as outlined in the Australian Code for the Responsible Conduct of Research.

#### Power and Sample Size

Power and sample size estimates were carried out using PS: Power and Sample Size Calculation (version 3.1.2; Vanderbilt University) [56], assuming a type 1 error probability of <.05, 2-tailed, and equal-sized groups. Power calculations were based on the primary end point and the change in the proportion of participants who accurately identified alcohol as a clear risk factor for breast cancer. On the basis of the data from our web-based survey of breast screening service consumers (n=391) conducted as part of the study’s formative evaluation, the proportion of participants identifying alcohol as a clear risk factor for breast cancer at baseline was estimated to be 22%. On the basis of a related study examining the effects of a public health campaign on awareness of the alcohol-breast cancer link [57], the response rate for participants receiving an alcohol brief intervention was estimated to be ≥12% greater at 34% at 4 weeks postrandomization. Accounting for 20% attrition at the follow-up, a sample of at least 548 participants (274 per arm) was estimated to provide 80% power to reject the null hypothesis.

#### Statistical Analysis Plan

Data were collated, cleaned, and validated in a database that was locked before analysis. No interim analyses were planned to be conducted, and as no substantial harm was anticipated from this trial, no formal stopping guidelines were specified. All statistical tests were 2-tailed, with the α level set at .05, and analyses were conducted using the most appropriate procedures in Stata or R All randomized participants were included in the analyses (ie, intention-to-treat) for primary and secondary outcomes. Sociodemographic characteristics will be summarized and reported by intervention arm. Analyses examined change in outcomes over time (4 weeks, 12 weeks, or 4 and 12 weeks; pre-specified for each outcome) relative to baseline. The Generalized Linear Mixed Model approach with fixed effects for treatment and time, their interaction, and random effects for subjects and assessments within subjects was applied to examine the treatment effect of the intervention on all outcomes. For outcomes assessed at a single postrandomization time point, the models were equivalent to traditional logistic regression. For consumption outcomes, subgroup analyses were conducted with participants exceeding national guidelines for weekly consumption. Mixed models are robust to missingness under the assumptions of missing at random and missing completely at random, thereby maintaining analyses based on intention-to-treat principles. For outcomes with 2 time points, Cochran-Mantel-Haenszel tests were examined.

#### Trial Monitoring

The trial’s chief investigators performed the function of a Trial Steering Committee, because they had the technical expertise necessary to oversee all aspects of trial conduct (eg, monitor compliance with the protocol, provide ethical and clinical governance, provide standardized training and other means of quality control, and monitor trial arm fidelity). Given the minimal risks of harm associated with this brief intervention, a formal data-monitoring committee was not considered necessary. Regular liaison between the immediate research team and the principal investigator occurred to permit discussion of day-to-day trial progress and any potential concerns. The broader research team met intermittently to review the overall progress of the project and conduct the trial. Data audits were conducted at periodic intervals and were led by the trial manager.

#### Protocol Amendments

Protocol amendments were approved by the Eastern Health Human Research Ethics Committee. No major changes to the protocol were implemented without prior approval and there were no modifications to the intervention during the trial.

#### Adverse Events

The risk of harm or discomfort to participants during this trial was anticipated to be minor and transient, in line with the research team’s experience conducting brief alcohol intervention research (eg, minor distress responding to questions about alcohol consumption). The research team monitored possible adverse effects throughout the trial.

#### Participant Withdrawal and Discontinuation

All participants were advised that they had the right to withdraw consent at any time and without consequences. Withdrawal of consent could occur verbally or in written form (ie, email or text message correspondence), and participants could elect to remove all of their previously collected data or consent for further data collection. No further contact was initiated by the research team once the participants withdrew consent.

#### Ancillary and Posttrial Care

Participants were advised during enrollment and at the end of participating in their allocation intervention that they should continue to engage with health care as usual while participating in the study and to contact their primary care provider if their participation in this study raised concerns about any aspect of their health (ie, alcohol use, general health, breast cancer risk).

### Program Implementation Evaluation

#### Setting

Researchers conducting the process evaluation were based at Turning Point, with telephone or videoconferencing used to collect feedback from trial participants and breast screening staff.

#### Participants

The first component of the process evaluation comprised quantitative feedback about the health information received (Health4Her and control) and its implementation from the 497 women who participated in the 12-week follow-up assessment and responded to the program evaluation questions. Qualitative data about the Health4Her intervention and its implementation were collected from in-depth semistructured telephone interviews conducted with 30 participants after trial. A nested sample design was used to select a subsample of participants for the qualitative interviews [58]. All women were asked if they would be interested in being contacted for an optional qualitative telephone interview. From those who consented to participate in this extra component of the study, systematic random sampling was used to contact participants who (1) were randomized to the active arm and (2) reported drinking alcohol at baseline to arrange an interview. Of this sample, 9 women were purposively sampled from participants who were drinking above the Australian Alcohol Guidelines at baseline assessment and who had consented to the optional interview.

Staff feedback about the intervention and implementation was obtained via 3 videoconferencing focus groups with 11 Maroondah BreastScreen staff members (managers=2, radiographers=6, and reception staff=3) held at the completion of the trial. All staff members who were interested in participating in a focus group and who were available on the day a focus group was being run were able to take part.

#### Data Collection

Applicable elements of the RE-AIM framework [26] guided the development of the quantitative participant feedback schedule, the semistructured participant interview, and interpretation of participant feedback to understand the Health4Her intervention’s reach (eg, participants’ use of health information and reach of information beyond trial participants [distributed health literacy]), efficacy (eg, intervention acceptability and perceived benefits), and implementation (eg, intervention delivered via iPad). Some elements of the CFIR [25] were also used to understand the characteristics of the intervention (eg, format and delivery) and outer setting (eg, existing health campaigns and intervention needs) from the perspectives of participants to identify factors influencing successful implementation.

Applicable elements of the CFIR [25] were used to guide the development of the semistructured staff focus group schedule and interpretation of staff feedback to understand the outer setting (eg, client needs and resources) and inner setting (eg, Maroondah BreastScreen culture, implementation climate, compatibility, and resources). Elements of the RE-AIM framework [26] were also used to understand reach and efficacy relative to staff facilitating intervention delivery (eg, letting women know about the intervention in advance), adoption (eg, factors predicted to affect the uptake of the Health4Her intervention at other breast screening services), and maintenance (eg, factors that facilitate long-term uptake of the Health4Her intervention).

Guided by the RE-AIM framework [26], the process evaluation also included the evaluation of trial and service administrative data collected during implementation (eg, intervention reach and uptake, such as the rate at which women participate in the trial of women invited to participate, reasons for nonparticipation when available, participant characteristics including health inequity factors [culturally and linguistically diverse background; lesbian, gay, bisexual, transgender, intersex, queer, and other LGBTIQ+ status; experiencing disability; low education; and living alone], intervention reach beyond trial participants [distributed health literacy], and COVID-19 impact on operations).

#### Data Analyses

Summary data from quantitative trial and service data and participant feedback were expressed as counts and proportions or means and SDs. For participant and staff qualitative data, interviews and focus groups were audio-recorded and transcribed verbatim by a professional transcribing company and organized using NVivo 20 according to the relevant domains of the RE-AIM and CFIR frameworks. Deductive thematic analysis was conducted using the framework method [33].

### Ethics Approval

This research was conducted in accordance with the Declaration of Helsinki, the 2007 National Health and Medical Research Council National Statement on Ethical Conduct in Human Research (updated 2018), the 2000 Note for Guidance on Good Clinical Practice (CPMP/ICH/135/95), and the International Council for Harmonisation Good Clinical Practice Guidelines. This protocol and informed consent processes for each element of the research were approved by the Eastern Health Human Research Ethics Committee (LR19-011-50551) and the Monash University Human Research Ethics Committee (21,395). The trial was preregistered with ClinicalTrials.gov (NCT04715516) on January 20, 2021.

## Results

This research was funded in March 2019 (Eastern Health Foundation Research and Innovation Grant) and May 2019 (VicHealth Impact Research Grant). Data collection for the formative evaluation was conducted from January to April 2020 (retrospective analysis of alcohol consumption data from Lifepool, n=49,240; web-based survey with breast screening service consumers, n=391; focus group and interviews with breast screening service consumers, n=31). Trial recruitment was conducted from February to August 2021 (n=588), with follow-up data collection finalized in December 2021. Quantitative process evaluation data were collected during trial implementation, and the collection of participant and staff feedback was finalized in December 2021 (participant quantitative feedback, n=588; participant qualitative feedback, n=30; and breast screening site staff feedback, n=11). We anticipate that the results of the retrospective analysis of alcohol consumption data from Lifepool (part of the mixed methods formative evaluation) will be published in March 2023. We anticipate that the results of this RCT will be published in March 2023. The program evaluation (comprising the preimplementation web-based survey and focus groups and interviews with subsets of breast screening service consumers and mixed methods process evaluation using trial and site administrative data and feedback from trial participants and breast screening site staff) is also planned for publication.

## Discussion

### Anticipated Findings

Previous research has found brief alcohol interventions to be effective in increasing alcohol-related knowledge and reducing alcohol consumption in primary care and other settings [34,59]. This is the first known hybrid effectiveness-implementation trial to examine the benefits of a novel, tailored alcohol brief intervention among women attending the breast screening setting. The study design permits an extensive mixed methods program evaluation alongside a RCT to provide a sound understanding of the factors affecting implementation of the Health4Her intervention to predict and facilitate uptake in practice. The RCT is anticipated to provide evidence that Health4Her intervention increases the proportion of women accurately identifying alcohol as a breast cancer risk factor, improving alcohol literacy more broadly (ie, increasing the proportion of women accurately identifying the amount of alcohol in an Australian standard drink, the number of standard drinks in an average restaurant serve of red wine, and the maximum number of standard drinks per week recommended by current Australian Alcohol Guidelines), and reducing alcohol consumption. The breast screening appointment can act as a window of opportunity to increase engagement with breast cancer risk reduction information; this research will generate new substantial knowledge on the alcohol consumption and alcohol literacy needs of this population and the extent to which these can be addressed using a low-cost, scalable intervention, with the potential to contribute to the prevention of alcohol-attributable breast cancer and the related burden of disease and quality of life among this population.

### Strengths and Limitations

The hybrid type II effectiveness-implementation trial design, guided by applicable elements of the RE-AIM [26] and CFIR frameworks [25], represents a key strength of this study. The integration of quantitative and qualitative data occurring at each level of the implementation evaluation [60] will build upon the outcomes of the RCT to provide real-world indicators of program implementation, to accelerate the translation of this research into practice. The RCT used a double-blind design; a typical limitation of brief alcohol intervention studies with hazardous drinkers in primary care settings is the bias introduced by difficulties with participant blinding [61]. One limitation of this research is the risk of assessment reactivity, where mere exposure to alcohol questions prompts awareness and behavior self-regulation in the trial’s control group and can bias trial results toward the null and lead to underestimates of true intervention effects. In the design of the RCT, alcohol knowledge and consumption outcomes were interspersed with general lifestyle questions to minimize the salience of the trial’s alcohol focus. However, control group assessment reactivity may still occur. An additional limitation is that this initial trial was powered to identify a change in knowledge of alcohol as a breast cancer risk factor and not alcohol consumption per se. Still, this study will provide preliminary data on consumption end points, rates of missing data, and participant attrition, providing important estimates for future scaled-up research.

### Conclusions

The benefits of brief alcohol interventions have been demonstrated in primary care and other settings; however, their effectiveness and implementation in the breast screening setting remain to be explored. The Health4Her intervention, targeting alcohol literacy and harmful consumption among women attending the breast screening setting, has huge potential for scalability and is particularly relevant considering the increasing prevalence of risky drinking among middle- to older-age women and strong evidence that even very light alcohol consumption increases breast cancer risk. This study will evaluate both the effectiveness and implementation of Health4Her intervention to predict and facilitate uptake in breast screening services.

### Dissemination Plan

The findings of this research will be disseminated to funding bodies and other relevant stakeholders, published in peer-reviewed journals, presented at national and international conferences, and presented via public talks and media and social media.

## Appendix

Multimedia Appendix 1 Standard Protocol Items: Recommendations for Interventional Trials (SPIRIT) guidelines.

Multimedia Appendix 2 Template for Intervention Description and Replication (TIDieR) checklist.

## Abbreviations

CFIR: Consolidated Framework for Implementation Research
LGBTIQ+: lesbian, gay, bisexual, transgender, intersex, queer, and other
RCT: randomized controlled trial
RE-AIM: Reach, Effectiveness, Adoption, Implementation, and Maintenance
REDCap: Research Electronic Data Capture

## Data Availability

Only approved members of the research team had access to the final trial data set. Data were not shared, as there was no participant permission or ethics approval to do so, other than for related projects conducted by the research team.
